# Epidemiology of antimicrobial-resistant *Escherichia coli* carriage in sympatric humans and livestock in a rapidly urbanizing city

**DOI:** 10.1016/j.ijantimicag.2019.08.014

**Published:** 2019-11

**Authors:** Dishon Muloi, John Kiiru, Melissa J. Ward, James M. Hassell, Judy M. Bettridge, Timothy P. Robinson, Bram A.D. van Bunnik, Margo Chase-Topping, Gail Robertson, Amy B. Pedersen, Eric M. Fèvre, Mark E.J. Woolhouse, Erastus K. Kang'ethe, Samuel Kariuki

**Affiliations:** a Usher Institute of Population Health Sciences and Informatics, University of Edinburgh, Edinburgh, UK; b Centre for Immunity, Infection and Evolution, University of Edinburgh, Edinburgh, UK; c Institute of Infection and Global Health, University of Liverpool, Liverpool, UK; d International Livestock Research Institute, Nairobi, Kenya; e Nuffield Department of Clinical Medicine, University of Oxford, John Radcliffe Hospital, Oxford, UK; f Animal Production and Health Division, Food and Agriculture Organization of the United Nations, Rome, Italy; g Centre for Microbiology Research, Kenya Medical Research Institute, Nairobi, Kenya; h University of Nairobi, Nairobi, Kenya; i Institute of Evolutionary Biology, School of Biological Sciences, University of Edinburgh, Edinburgh, UK; j School of Mathematics, University of Edinburgh, Edinburgh, UK

**Keywords:** Antibiotic resistance, AMR, *Escherichia coli*, One Health, Surveillance

## Abstract

There are substantial limitations in understanding of the distribution of antimicrobial resistance (AMR) in humans and livestock in developing countries. This papers present the results of an epidemiological study examining patterns of AMR in *Escherichia coli* isolates circulating in sympatric human (*n* = 321) and livestock (*n* = 633) samples from 99 households across Nairobi, Kenya. *E. coli* isolates were tested for susceptibility to 13 antimicrobial drugs representing nine antibiotic classes. High rates of AMR were detected, with 47.6% and 21.1% of isolates displaying resistance to three or more and five or more antibiotic classes, respectively. Human isolates showed higher levels of resistance to sulfonamides, trimethoprim, aminoglycosides and penicillins compared with livestock (*P*<0.01), while poultry isolates were more resistant to tetracyclines (*P* = 0.01) compared with humans. The most common co-resistant phenotype observed was to tetracyclines, streptomycin and trimethoprim (30.5%). At the household level, AMR carriage in humans was associated with human density (*P*<0.01) and the presence of livestock manure (*P* = 0.03), but keeping livestock had no influence on human AMR carriage (*P*>0.05). These findings revealed a high prevalence of AMR *E. coli* circulating in healthy humans and livestock in Nairobi, with no evidence to suggest that keeping livestock, when treated as a single risk factor, contributed significantly to the burden of AMR in humans, although the presence of livestock waste was significant. These results provide an understanding of the broader epidemiology of AMR in complex and interconnected urban environments.

## Highlights

• First study of antimicrobial resistance (AMR) phenotypes in humans and urban livestock.
• Highest AMR carriage in humans, pigs and poultry.
• AMR more common in larger households.
• Urban livestock keeping is not a risk factor for AMR in humans.
• Use of animal manure affects the risk factor of AMR in humans.

## 1 Introduction

Antimicrobial resistance (AMR) in bacteria is regarded as one of the most serious public health threats of this century [1], [2], [3]. Over the last decade, increasing levels of resistance to clinically relevant antibiotics – including carbapenems [4] and colistin [5], which are considered antibiotics of last resort – have been reported in both human and animal populations.

Although *Escherichia coli* can be a harmless gut commensal, some pathogenic strains can cause life-threatening bloodstream infections and common illnesses, such as urinary tract infections [6]. *E. coli* can also cause disease in animals, leading to severe economic losses due to mortality and morbidity [7]. Recently, *E. coli* was categorized by the World Health Organization as a priority pathogen due to its widespread antibiotic resistance [8].

Livestock have been implicated as a reservoir for AMR bacteria that may spread to humans, with keeping livestock widely believed to be a risk factor for AMR in humans [9,10]. However, quantitative evidence describing the role of livestock in the emergence and transmission of AMR bacteria to human populations is lacking [11], particularly in low- and middle-income countries (LMICs) [12]. In the absence of routine surveillance of AMR in most LMICs, understanding the epidemiology of AMR is key to developing effective strategies to target a reduction in the emergence and spread of resistance in the future.

To date, studies investigating the epidemiology of AMR have tended to focus on either human or livestock populations without making comparisons of resistance between the two populations. A recent systematic review [11] of studies investigating the link of AMR *E. coli* between humans and livestock found only 22 studies of spatiotemporally-related isolates from human and livestock populations, just six of which were conducted in LMICs. Notably, none of these studies considered urban livestock, which are of increasing importance, particularly in LMIC settings [13], and may contribute to the maintenance of zoonotic bacteria and AMR in the complex urban environment [14].

This study focused on the role of keeping livestock as a potentially high-risk urban interface for AMR transmission between humans and livestock in urban Nairobi. Nairobi is a rapidly growing city where livestock are commonly kept within household compounds, bringing them into close contact with people. *E. coli* is an ideal organism to study the spread of AMR in this complex environment, as it is a ubiquitous commensal in both livestock and humans but with a wide range of resistance phenotypes.

This paper reports the results from the first study to characterize the patterns and epidemiology of antibiotic-resistant *E. coli* from cohabiting human and livestock populations in a low-resource urban setting. At the scale of individual households, the role of livestock is explored as a risk factor for AMR carriage in humans, hence providing insight into the pathways of AMR transfer.

## 2 Methods

### 2.1 Study design

A cross-sectional study targeting sympatric human and livestock populations in Nairobi, Kenya was carried out from August 2015 to October 2016 as part of the Urban Zoo Project [15]. Briefly, Nairobi was stratified into administrative sublocations according to socio-economic status, identifying 70 possible sublocations. Thirty-three sublocations were chosen with the aim of maximizing spatial distribution and socio-economic diversity, and attempting to capture the diversity of livestock-keeping practices across the city [15]. For each sublocation, three households – two that kept livestock [small livestock only (poultry, rabbits and goats) and large livestock (cattle and pigs) with or without small livestock] and one that did not keep livestock – were selected at random within the dominant housing type.

**Fig. 1:**
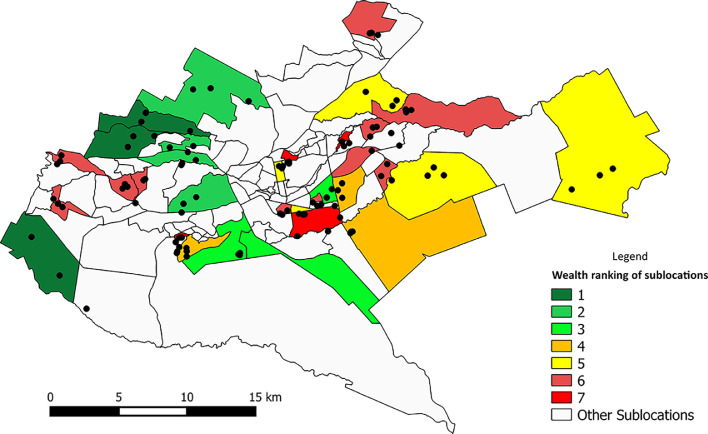
Map of Nairobi, Kenya indicating the location of the sampled households (black dots) and 33 sublocations (coloured by wealth category; 1, wealthy; 7, poor).

In total, 99 households were involved in the study (Fig. 1). The design of the study is explained in detail in the online supplementary material.

### 2.2 Sample collection and antimicrobial susceptibility testing

In each household, a questionnaire was used to collect data on household composition, socio-economic variables, livestock ownership, food consumption and medical history. Human and animal faecal samples were collected and transported on ice to one of two laboratories (University of Nairobi or Kenya Medical Research Institute) within 5 h of collection. Samples were enriched in buffered peptone water for 24 h, and thereafter plated on to eosin methylene blue agar (EMBA) and incubated for 24 h at 37 °C. One colony from each plate was selected and subcultured for a further 24 h on a second round of EMBA. Subsequently, one purified colony from each plate was selected at random (hereafter referred to as an ‘isolate’), and confirmed as *E. coli* by biochemical testing using triple sugar iron agar, Simmon's citrate agar and motility-indole-lysine media.

Antimicrobial susceptibility testing for 13 antibiotics – ampicillin (10 µg/mL), amoxicillin-clavulanic acid (30 µg/mL), cefepime (30 µg/mL), cefotaxime (30 µg/mL), ceftazidime (30 µg/mL), chloramphenicol (30 µg/mL), ciprofloxacin (5 µg/mL), gentamicin (10 µg/mL), nalidixic acid (30 µg/mL), streptomycin (25 µg/mL), sulfamethoxazole (30 µg/mL), tetracycline (30 µg/mL) and trimethoprim (2.5 µg/mL) – that are frequently used in either/both veterinary and/or human medicine in Kenya was undertaken using the Kirby–Bauer disc diffusion method (Oxoid Ltd, Basingstoke, UK). Standardized protocols were used, in which antibiotic discs were dispensed on to bacteria-containing agar plates and incubated for a maximum of 18 h at 35°C. *E. coli* ATCC 25922 was used as a quality control of the susceptibility tests.

Clinical and Laboratory Standards Institute interpretive criteria for Enterobacteriaceae [16] were used to determine breakpoints for classifying isolates as either susceptible (‘susceptible’ or ‘intermediate’) or non-susceptible (‘resistant’) for 11 of the 13 drugs. For tetracycline and trimethoprim, isolates were classified as resistant or susceptible because examination of the distributions of the zones of inhibition showed populations of isolates with distinct phenotypic resistance patterns (see Table S1 in online supplementary material). To describe multi-drug patterns, the overall resistance profile was calculated by combining the resistance phenotype to each individual class, and thus antibiogram length (hereafter also referred to as ‘AMR carriage’) is the total number of antibiotic classes to which an isolate was phenotypically resistant.

### 2.3 Statistical analysis

The distribution of resistance phenotypes between hosts was calculated using Chi-squared tests (humans and livestock) and a one-way analysis of variance (ANOVA; human vs different livestock groups). Tukey's multiple-comparison test was performed *post hoc* for pairwise comparisons between groups, and *P*-values <0.05 were considered significant.

Generalized linear mixed models (GLMMs), implemented in R package ‘lme4’ [17], with antibiogram length as the dependent variable were used to test whether AMR carriage differed between host groups. To investigate the co-occurrence of AMR phenotypes, a pairwise co-occurrence matrix (presence and absence) of the phenotypes was constructed using polycor package [18] in R and the co-occurrence relationships were visualized using corrplot [19]. A correlation between two AMR phenotypes was considered statistically significant if the *P*-value (adjusted for multiple testing using Bonferroni's correction) was <0.05.

To investigate finer scale household-level risk factors for AMR carriage in humans, a Poisson-distributed GLMM was fitted with the counts of resistance phenotypes (antibiogram length) as the response variable. Model explanatory variables were human density (count of people in a household as a function of household area) and types of livestock kept by the household (small livestock only, large livestock with or without small livestock, and no livestock). Additionally, for households that kept livestock, a separate Poisson-distributed GLMM was fitted to investigate the effect of human density and manure disposal practises (manure disposed in the household compound or outside) on human antibiogram length. Separate models were fitted for the most prevalent AMR phenotypes (tetracyclines, aminoglycosides, sulfonamides, penicillins and trimethoprim).

To account for the nested (or hierarchical) nature of the sampling design, household site (*n* = 99), sublocation (*n* = 33) and wealth category (*n* = 7) were included as random factors. Further details of data exploration and statistical models are given in the online supplementary material.

## 3 Results

In total, 954 isolates composed of 321 human and 633 livestock *E. coli* isolates were analysed. The number of isolates obtained from each source is presented in Table 1.

**Table 1:** Number of human and livestock isolates collected from 99 households in Nairobi, Kenya (2015–2016).

Source	Number of isolates	% of isolates
Human	321	33.7
Livestock:		
Poultry	345	36.2
Bovine	64	6.7
Goat	132	13.8
Pig	51	5.3
Rabbit	41	4.3

### 3.1 Patterns of AMR in humans and livestock

**Fig. 2:**
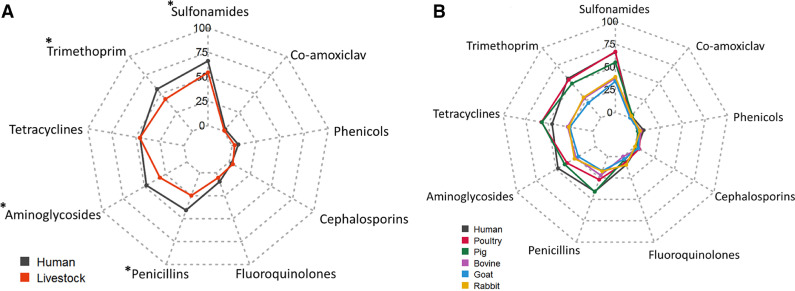
Radar charts showing percentages of *Escherichia coli* isolates resistant to nine antibiotic classes. (a) Human (*n* = 321) and livestock (*n* = 633). (b) Human and different livestock species (poultry, pig, bovine, goat and rabbit). Asterisks denote significant differences between carriage of this particular resistance phenotype in livestock and humans.

The most common resistance phenotypes (>40% of resistant isolates) were to sulfonamides, trimethoprim, tetracyclines and aminoglycosides. A smaller percentage of isolates (<10%) were resistant to amoxicillin/clavulanic acid, cephalosporins, phenicols and fluoroquinolones (Table 2 and Fig. 2). The distribution of resistance to the individual drugs tested is given in Table S2 (see online supplementary material).

**Table 2:** Percentages of *Escherichia coli* isolates resistant to different antibiotic classes classified by host type (human or livestock).

Antibiotic category	Overall (*n* = 954)	Human (*n* = 321)	Livestock (*n* = 633)	Adj. *P* value
Sulfonamides	58.2	66	54.2	0.005
Aminoglycosides	37.1	47.7	31.8	<0.001
Trimethoprim	47.3	56.1	42.8	0.001
Tetracyclines	45.7	45.5	45.8	NS
Penicillins	30.2	40.8	24.8	<0.001
β-lactam (co-amoxiclav)	1.5	2.5	0.95	NS
Phenicols	4.0	6.5	2.69	NS
Cephalosporins	3.8	2.8	4.27	NS
Fluoroquinolones	6.8	9.7	5.37	NS

NS, not significant.

Numbers show percentages of isolates classified as resistant based on the zone of inhibition. Categorical interpretation is based on breakpoints used as described in the text.

When analysed by host, human isolates were more commonly resistant to each of the individual antibiotic classes than those of animal origin. Of 321 human isolates, >40% were resistant to sulfonamides, trimethoprim, aminoglycosides and tetracyclines. Of 633 livestock isolates, >40% of isolates were resistant to sulfonamides, tetracyclines and trimethoprim. For both human and livestock isolates, <10% of isolates were resistant to phenicols, fluoroquinolones, cephalosporins and beta-lactams. Resistance to penicillins, aminoglycosides, sulfonamides and trimethoprim was significantly more common in humans than in livestock (*P*<0.01, Chi-squared test; Table 2 and Fig. 2a).

The prevalence of resistance to penicillins, tetracyclines, aminoglycosides, sulfonamides and trimethoprim varied significantly between humans and livestock stratified by taxonomic groups (poultry, pigs, rabbits, bovines and goats; Tukey's post-hoc test). Humans were more likely to carry *E. coli* resistant to penicillins, aminoglycoside, sulfonamides and trimethoprim than all species of livestock (*P*<0.05, one-way ANOVA with Tukey's multiple-comparison test). Conversely, poultry were more likely to carry isolates resistant to tetracyclines than humans (Fig. 2b and Fig. S1, see online supplementary material).

**Fig. 3:**
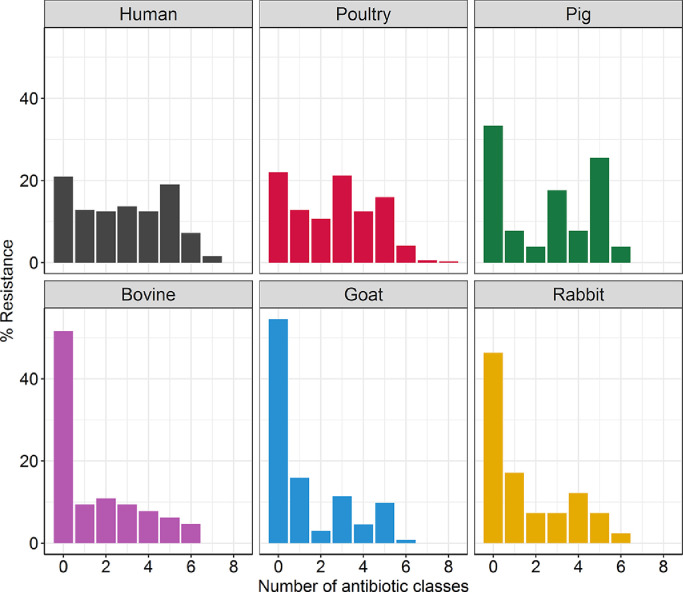
Distribution of multi-drug resistance patterns among *Escherichia coli* isolates obtained from humans (*n* = 321), poultry (*n* = 345), pigs (*n* = 51), bovines (*n* = 64), goats (*n* = 132) and rabbits (*n* = 41) in Nairobi, Kenya.

Overall, 284 (29.7%) isolates were susceptible to all 13 antibiotics tested (nine antibiotic classes). The proportion of pan-susceptible isolates was significantly higher among livestock isolates (*n* = 217/633, 34.3%) than human isolates (*n* = 67/321, 20.9%) (χ^2^ = 17.6, *P*<0.01, Chi-squared test). Of the 217 pan-susceptible livestock isolates, 22% of poultry isolates (*n* = 76), 51.6% of bovine isolates (*n* = 33), 33.3% of pig isolates (*n* = 17), 54.6% of goat isolates (*n* = 72) and 46.3% of rabbit isolates (*n* = 19) were pan-susceptible. Across both human and livestock isolates, 404 (47.6%) and 201 (21.1%) isolates were resistant to three or more and five or more antibiotic classes, respectively. Eight isolates (0.8%) showed resistance to seven or more antibiotic classes tested; five (1.6%) from humans and three (0.9%) from poultry (Fig. 3).

Antibiogram length (i.e. the total number of antibiotic classes to which an isolate is resistant) was significantly higher in humans than in livestock [odds ratio (OR) = 1.14, 95% confidence interval (CI) 0.68–0.81, *P*<0.01, marginal *R*^2^ = 0.041, GLMM]. However, when studied in more detail, antibiogram lengths in human isolates were similar to those from pigs and poultry (*P*>0.05, marginal *R*^2^ = 0.151, GLMM) but significantly higher than those from bovines, goats and rabbits (*P*<0.05, marginal *R*^2^ = 0.151, GLMM) (Table 3 and Fig. 3).

**Table 3:** Results of a Poisson generalized linear mixed model examining the likelihood of antimicrobial resistance carriage within different host groups.

	No. of isolates	Estimate	Standard error	*P* value
Human	321	Reference	Reference	Reference
Livestock	633	−0.13	0.16	<0.01
Bovine	64	−0.28	0.14	0.03
Poultry	345	−0.08	0.05	NS
Pigs	51	0.08	0.11	NS
Rabbits	41	−0.37	0.16	0.02
Goats	132	−0.48	0.11	<0.01

NS, not significant.

Human is used as the reference level.

**Fig. 4:**
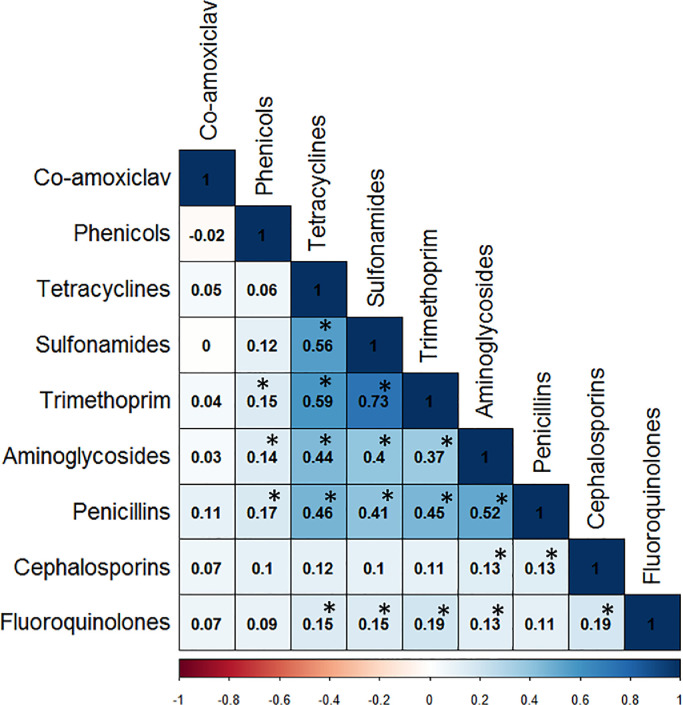
Heat map representing correlations among antimicrobial resistance phenotypes across human (*n* = 321) and livestock (*n* = 633) *Escherichia coli* isolates. The boldness of the colour represents the strength of the relationship between phenotypes, with stronger correlations having bolder colours. Numbers within boxes represent correlation coefficient (*r*) values. Asterisks indicate statistically significant correlations (*P*<0.05). The scale bar indicates whether the correlation between phenotypes is positive (closer to 1, dark blue) or negative (closer to −1, dark red).

Examination of the similarity of *E. coli* antibiograms from human and livestock isolates revealed 84 distinct profiles: 30 in livestock, 19 in humans and 35 common to both (Table S4, see online supplementary material). Using a co-occurrence analysis based on a statistically significant (*P*<0.05) correlation coefficient (ρ>0.5), a tetracycline-sulfonamide-trimethoprim cluster was identified (Fig. 4). This co-resistance was identified in 340 isolates (30.5%): 115 (35.8%) humans and 225 (35.5%) livestock – 156 (45.2%) poultry, 24 (47.1%) pigs, nine (22.0%) rabbits, 14 (21.9%) bovines and 22 (16.7%) goats. There were no significant differences in the distribution of this profile between human and the other host groups (χ^2^<0.01, *P*>0.98, Chi-squared test). Further, denoting multi-resistance, this cluster was commonly associated with resistance to aminoglycoside and penicillins.

### 3.2 AMR exchange between humans and livestock at the household level

**Fig. 5:**
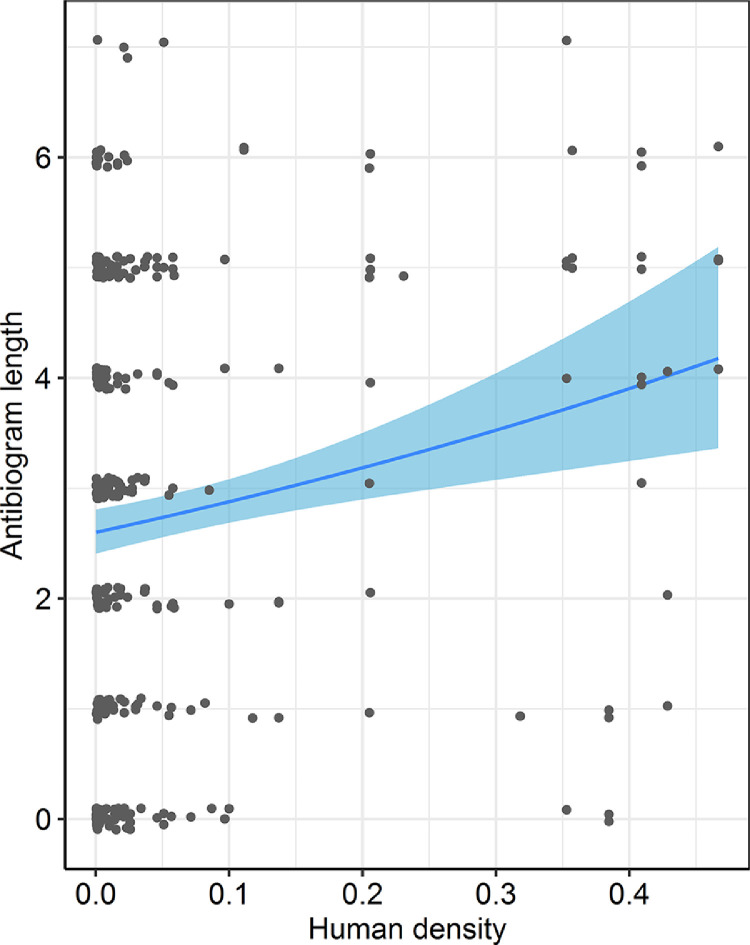
Fit of a Poisson generalized linear mixed effects model showing how increasing human density in a household influences the antibiogram length in humans. All other covariates in the models are kept constant. Shading on either side of each line represents 95% confidence intervals. Points have been jittered for clarity.

In any given household, no evidence was found to indicate that the presence of livestock increased the risk of human AMR carriage (large livestock OR = 0.94, 95% CI 0.72–1.22, *P* = 0.24; small livestock OR = 1.04, 95% CI 0.82–1.30, *P* = 0.94, marginal *R*^2^ = 0.3, GLMM) (Table 4). However, human antibiogram length increased with human density (OR = 1.26, 95% CI 1.08–1.47, *P* = 0.003, marginal *R*^2^ = 0.3, GLMM) (Fig. 5). The impact of keeping livestock on human AMR carriage was potentially influenced by disposal practices of animal manure: keeping manure inside the household perimeter, compared with disposing of it externally, was associated with greater human antibiogram length (OR = 1.29, 95% CI 1.02–1.63, *P* = 0.03, marginal *R*^2^ = 0.5, GLMM) (Table 4). These results were consistent when separate analyses were performed for the individual resistances (Table S3, see online supplementary material).

**Table 4:** Results of two generalized Poisson mixed models investigating household risk factors for antimicrobial resistance carriage (antibiogram length) in humans at the household level.

Model 1: Antibiogram length, humans in all households	Estimate	Standard error	*P* value
Human density	0.23	0.08	0.003
Large livestock (with or without small livestock)	−0.14	0.12	0.24
Small livestock only	0.0075	0.11	0.94


Model 2: Antibiogram length, humans in livestock-keeping households alone			
Human density	0.24	0.09	0.009
Manure in household	0.26	0.12	0.03

Households not keeping livestock used as the reference level in Model 1.

## 4 Discussion

This study applied ecological and epidemiological approaches to characterize the epidemiology of AMR *E. coli* isolates collected systematically from sympatric human and livestock populations in the rapidly developing urban landscape of Nairobi, Kenya.

Resistance to aminoglycosides, sulfonamides, tetracyclines, trimethoprim and penicillins was high in both humans and livestock, while resistance to cephalosporins and fluoroquinolones was low. These results are consistent with previous studies [20], [21], [22], [23] and may reflect, in part, the patterns of antibiotic use in human and animal health. However, background data on antimicrobial use in these populations is limited. The results indicating a high prevalence of AMR carriage are based on non-clinical isolates from humans and livestock.

When analysed by host, human isolates appeared to have a higher prevalence of AMR carriage compared with livestock isolates, with the exception of tetracyclines. In particular, the observed prevalence was significantly higher in four clinically relevant antibiotic classes: penicillins, sulphonamides, trimethoprim and aminoglycosides. A possible explanation for this variation in AMR carriage is that it relates to variation in antibiotic use between these populations. Although antibiotics are used extensively in both human and livestock populations, previous studies have shown that frequency of use of antibiotics is higher in human medicine than in livestock medicine, especially in resource-poor settings [24,25]. Similarly, in community settings where over-the-counter access to drugs is common, it is likely that humans have access to a broader range of antibiotics, either through self-medication or inappropriate prescribing; common practices in many LMICs [26,27]. Likewise, in such settings, infections are commonly treated empirically (often using antibiotics) with limited microbiological investigations to ascertain the causal organism(s).

Although the use of chloramphenicol in food animals has been banned in Kenya [28], 3% resistance to this antibiotic in livestock was noted. This may be explained by the use of florfenicol, a fluorinated derivative of chloramphenicol, which shows some cross-resistance with chloramphenicol [29]. Similarly, the observed levels of resistance against ciprofloxacin (a quinolone antimicrobial not licensed for veterinary use) among livestock isolates is probably explained by cross-resistance with other quinolones used in veterinary medicine, such as enrofloxacin and norfloxacin.

At the household level, there is evidence of an intricate interplay between humans and livestock in relation to the development and transmission of AMR. This analysis revealed that human AMR carriage increased with number of occupants in a household, and that keeping manure inside the household compound was also significantly associated with AMR carriage in humans. In urban Nairobi, people live in a continuum of urban spaces with varying human and animal population densities, with the majority (>60%) of people living in slums [30,31]; environments characterized by small household areas and high population densities. Population density is an important factor in the population prevalence of AMR [32], and may, in part, be due to the significant correlation between overcrowding and high burden of infectious diseases more broadly [33]; an important driver of antibiotic use in resource-poor settings such as Nairobi. Similarly, high human populations within a household result in greater epidemiological connectivity, thus facilitating exchange of AMR bacteria and their AMR determinants. The number of urban dwellers in the majority of LMIC cities, including Nairobi, is projected to grow significantly in the near future [34]. While this urban demographic change is unfolding, disease burden is expected to burgeon, precipitating high use of antibiotics. For this reason, measures to curb the infectious diseases burden by public health policy makers, in part to reduce drug pressure on micro-organisms, are needed.

These results suggest that, at the household level, keeping livestock in and of itself does not add to the risk of acquisition or carriage of AMR bacteria in humans. However, given the multiple pathways of AMR exchange between humans and livestock [35], via the food chain or due to environmental pollution, it is possible that the direct effect of keeping livestock on levels of AMR in humans could be confounded by other factors not captured in this study. This study does, however, suggest that, whilst AMR carriage (antibiogram length) was not directly associated with the presence of livestock in the household, the impact of keeping livestock on human AMR carriage was mediated by some practices associated with keeping livestock, namely the presence or absence of animal manure in the household. These results support other studies that have identified animal manure as a reservoir of AMR bacteria and AMR determinants [36,37]. Importantly, amplification and persistence of AMR determinants such as AMR plasmids can take place in manure and be further disseminated to humans via cross-contamination pathways such as through exposed water and food [38], or via peri-domestic wildlife. Although there is still a lack of knowledge concerning the exact mechanism, particularly the genetic basis of transmission [39], strategies that limit AMR gene flow to and from manure (to humans) should be adopted. Such measures include safe disposal of manure from households, and manure pre-treatment prior to application on to crop farms where possible.

It is important to note that, while this analysis was not designed to address transmission of AMR bacteria and their AMR determinants, it is also plausible that clonal expansion could have played a role in the observed AMR patterns. The finding of 35 common AMR profiles in both human and livestock bacterial populations may, in part, reflect overlapping antibiotic usage patterns, acquisition of AMR from a shared source or clonal expansion. It is hypothesized that the finding that 30.5% (340/954) of all isolates contain a tetracycline-sulfonamide-trimethoprim cluster phenotype and that the pairwise correlations between these three antibiotic classes were very high is suggestive of a conjugative MDR plasmid circulating within the *E. coli* population in both human and livestock populations. AMR genes conferring resistance to tetracycline, sulfonamide and trimethoprim antibiotic classes are commonly associated with mobile genetic elements [40], and these elements play a pivotal role in dissemination of multi-drug resistance in *E. coli* isolates. Genetic data are required to validate the existence of mobile genetic elements, and determine whether AMR genes are being transferred across them.

Distinguishing molecular transmission of AMR from selection for AMR due to antibiotic use is challenging [11]. In particular, in an urban environment such as Nairobi, where human habitation, keeping livestock and food supply chains are interconnected [41], the relative contributions of the aforementioned drivers are difficult to quantify. At a finer scale, any study investigating the transmission of AMR between humans and livestock in low-resource settings needs to consider indirect transmission, rather than just direct animal-to-human and/or human-to-animal transmission. Whilst direct host-to-host transmission of AMR bacteria and AMR determinants may occur, in these intricate ecosystems, the role played by the wider environment (e.g. wildlife, soil and, in particular, hospital and farm effluents) in relation to acquisition of AMR from a common source may be vital.

## 5 Conclusion

This rigorously structured epidemiological study found a high prevalence of AMR *E. coli* carriage in livestock and humans outside the clinical setting across a developing country urban landscape, with no evidence that direct contact with livestock contributes to the burden of human AMR, but that indirect contact between livestock and humans does play a role. In LMIC urban ecosystems, the elevated prevalence of AMR in both human and livestock populations could be attributed to unregulated access to antibiotics, poor hygiene and sanitation, and waste management, which encourage the evolution and spread of AMR bacteria. These findings highlight a need for targeted surveillance strategies across various sectors, and for actors to address and design effective measures to curb AMR in these populations, both in Nairobi and in other similar urban landscapes. Further work is required to understand the ecology of genetic determinants of resistance, particularly the extent of the role that plasmids play in the dissemination and evolution of resistance traits in these human and livestock populations.

## Appendix Supplementary materials

Image, application 1
