# Supplementary material for: Epidemiology of antimicrobial-resistant Escherichia coli carriage in sympatric humans and livestock in a rapidly urbanizing city
Source: Int J Antimicrob Agents. 2019 Nov;54(5):531–7. doi: 10.1016/j.ijantimicag.2019.08.014 (PMC6839611; doi:10.1016/j.ijantimicag.2019.08.014)
Supplement: Supplementary file 1 [file mmc1.docx]

**Supplementary Material**

**Table S1.** Antibiotics tested (and the respective antibiotic classes) and interpretation of zone of inhibition (mm). * Custom breakpoints based on examination of the distributions of the zones of inhibition.

|  |  |  | **Breakpoints Used (mm)** | | |
| --- | --- | --- | --- | --- | --- |
| **Antibiotic class** | **Antibiotic** | **Disc Content** µ**g** | **Susceptible** | | **Resistant** |
| β-lactam antibiotic | Amoxicillin-clavulanic acid | 10 | ≥18 | <13 | |
| Penicillin | Ampicillin | 10 | ≥17 | <13 | |
| Cephalosporin |  |  |  |  | |
| Third-generation | Cefotaxime | 30 | ≥26 | <22 | |
| Third-generation | Ceftazidime | 30 | ≥21 | <17 | |
| Fourth-generation | Cefepime | 30 | ≥25 | <18 | |
| Fluoroquinolone | Ciprofloxacin | 10 | ≥21 | <15 | |
|  | Nalidixic acid | 30 | ≥19 | <13 | |
| Aminoglycoside | Streptomycin | 25 | ≥15 | <11 | |
|  | Gentamycin | 10 | ≥15 | <12 | |
| Phenicol | Chloramphenicol | 30 | ≥18 | <12 | |
| Sulfonamide | Sulfamethoxazole | 30 | ≥17 | <12 | |
| Folate pathway/acid inhibitor* | Trimethoprim | 2.5 | ≥16 | <10 | |
| Tetracycline* | Tetracycline | 30 | ≥15 | <11 | |

**Table S2.** Percentages of *E. coli* isolates resistant to a panel of 13 antibiotics classified by host type. Numbers show percentages of isolates classified as resistant based on the zone of inhibition. Definition of resistance is based on breakpoints used as described in the Material and Methods.

| Antibiotic class | Drug | Human  (n=321) | Poultry  (n=345) | Pig  (n=51) | Bovine  (n=64) | Goat  (n=132) | Rabbit  (n=41) |
| --- | --- | --- | --- | --- | --- | --- | --- |
| β-lactam | Co-amoxiclav | 2.5 | 0.6 | 3.9 | 1.6 | 0 | 2.4 |
| Penicillins | Ampicillin | 40.8 | 27 | 41.2 | 21.9 | 16.7 | 17.1 |
| Phenicols | Chloramphenicol | 6.5 | 3.5 | 0 | 3.1 | 1.5 | 2.4 |
| Cephalosporins | Ceftazidime | 1.6 | 1.4 | 2 | 0 | 1.5 | 0 |
|  | Cefotaxime | 2.2 | 4.3 | 2 | 4.7 | 3.8 | 0 |
|  | Cefepime | 2.2 | 0.9 | 2 | 0 | 0.8 | 0 |
| Fluoroquinolones | Ciprofloxacin | 2.2 | 2.6 | 0 | 0 | 1.5 | 4.9 |
|  | Nalidixic acid | 9.7 | 6.7 | 3.9 | 0 | 3 | 9.8 |
| Sulfonamides | Sulfamethoxazole | 66 | 66.7 | 54.9 | 37.5 | 34.1 | 39 |
| Tetracyclines | Tetracycline | 45.5 | 57.4 | 56.9 | 28.1 | 25.8 | 26.8 |
| Aminoglycosides | Streptomycin | 47 | 35.4 | 39.2 | 25 | 22 | 26.8 |
|  | Gentamicin | 2.5 | 2.6 | 0 | 1.6 | 1.5 | 4.9 |
| Trimethoprim | Trimethoprim | 56.1 | 54.5 | 49 | 28.1 | 21.2 | 29.3 |


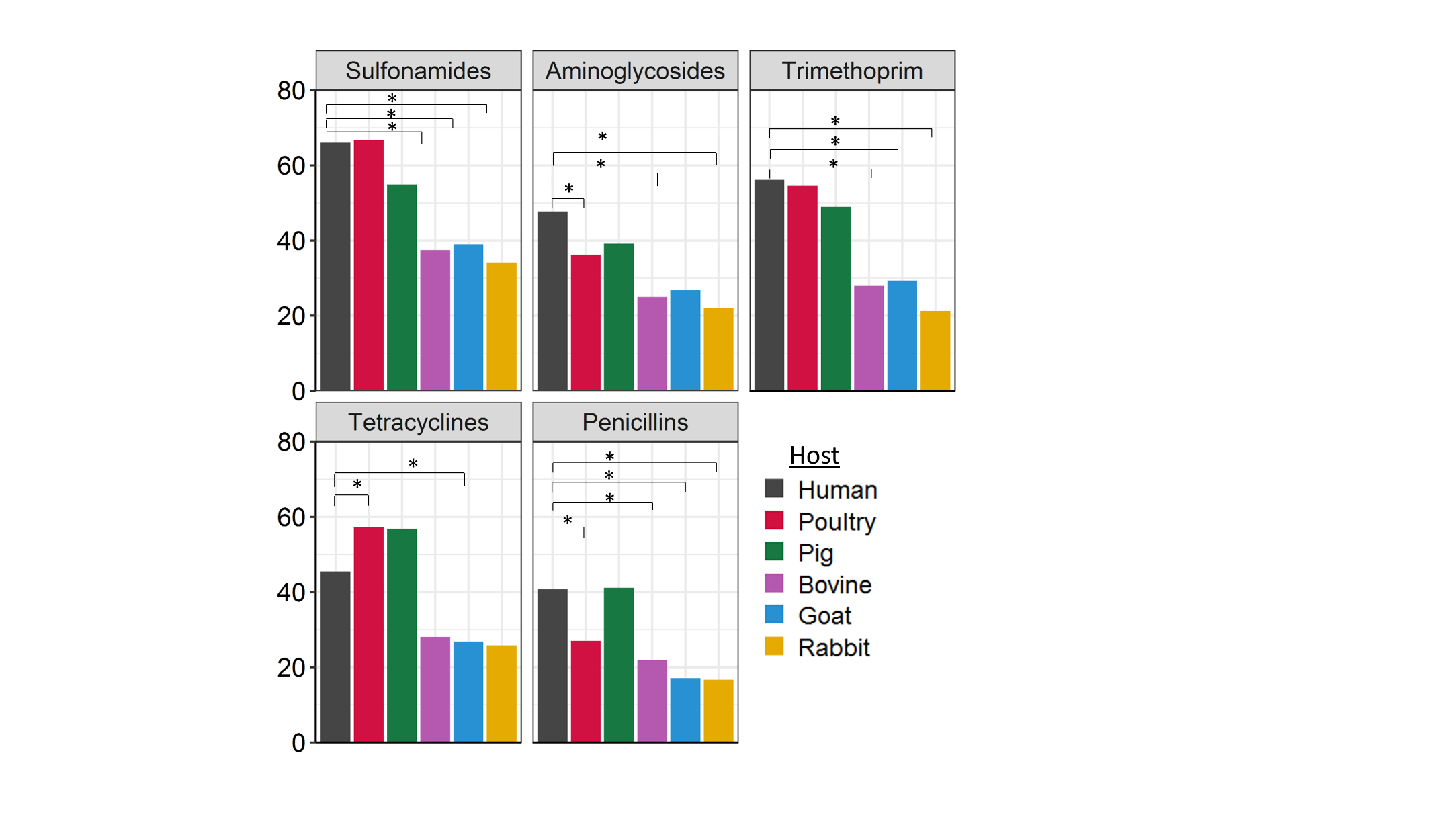
**Fig. S1.** Percentages of *E. coli* isolates resistant to five antibiotic classes by source type: human (n=321), poultry (n=345), pig (n=51), bovine (n=64), goat (n=132), and rabbit (n=41). P values are from a post-hoc Tukey’s test. Only antibiotic classes (5/9) with statistically significant differences in the percentages of resistance between the sources types are shown.

**Table S3.** Results of separate Poisson generalised linear mixed models investigating household risk factors for individual resistances by class in humans at the household level. Households not keeping livestock and having no manure in the household were used as the reference level in all models. Given the low prevalence of resistance (<5%) to co-amoxiclav, phenicols, cephalosporins and fluoroquinolones in our dataset, we did not include these in this analysis.

| **Variable** | **Estimate** | **Standard error** | **P value** |
| --- | --- | --- | --- |
| **Model 1: Antibiogram length, humans in all households** |  |  |  |
| **Tetracyclines** | | | |
| Human density | 0.6432 | 0.2069 | 0.00188 |
| Large livestock (with or without small livestock) | 0.1249 | 0.3068 | 0.68405 |
| Small livestock only | 0.4232 | 0.2849 | 0.13737 |
| **Aminoglycosides** | | | |
| Human density | 0.6329 | 0.2213 | 0.00424 |
| Large livestock (with or without small livestock) | -0.1438 | 0.324 | 0.65711 |
| Small livestock only | -0.2107 | 0.3011 | 0.48398 |
| **Sulfonamides** | | | |
| Human density | 0.61873 | 0.22858 | 0.00679 |
| Large livestock (with or without small livestock) | 0.07985 | 0.31112 | 0.79745 |
| Small livestock only | 0.55916 | 0.29782 | 0.06045 |
| **Penicillins** | | | |
| Human density | 0.7688 | 0.2304 | 0.000847 |
| Large livestock (with or without small livestock) | -0.639 | 0.3371 | 0.058015 |
| Small livestock only | -0.1738 | 0.3048 | 0.568543 |
| **Trimethoprim** | | | |
| Human density | 0.3728 | 0.2054 | 0.0695 |
| Large livestock (with or without small livestock) | -0.2668 | 0.3013 | 0.3759 |
| Small livestock only | 0.1893 | 0.2819 | 0.5019 |
| **Model 2: Antibiogram length, humans in livestock keeping household only** |  |  |  |
| **Tetracyclines** | | | |
| Human density | 0.7179 | 0.2599 | 0.005733 |
| Manure in household | 0.3683 | 0.313 | 0.239321 |
| **Sulfonamides** | | | |
| Human density | 0.5369 | 0.2866 | 0.061 |
| Manure in household | 0.3933 | 0.3142 | 0.211 |
| **Aminoglycosides** | | | |
| Human density | 0.6915 | 0.2926 | 0.01812 |
| Manure in household | 0.1996 | 0.3507 | 0.56919 |
| **Penicillins** | | | |
| Human density | 0.6728 | 0.2571 | 0.00886 |
| Manure in household | 0.566 | 0.3384 | 0.09435 |
| **Trimethoprim** | | | |
| Human density | 0.2467 | 0.2508 | 0.325 |
| Manure in household | 0.456 | 0.3049 | 0.135 |

**Table S4.** Profile composition and number of *E. coli* isolates from human and animal in Nairobi, Kenya. Abbreviations: Cephalosporins (Ceph), Aminoglycosides (Amg), Phenicols (Phn), Tetracyclines (Tet), Penicillins (Pcn), Penicillin lactams (Amc), Folate inhibitors (Tmp), Sulfonamides (Sul) and Fluoroquinolones (Fq).

| **Profile composition** | **Human** | **Poultry** | **Bovine** | **Goat** | **Pig** | **Rabbit** |
| --- | --- | --- | --- | --- | --- | --- |
| Pansusceptible | 67 | 76 | 33 | 72 | 17 | 19 |
| Amc | 0 | 0 | 1 | 0 | 1 | 0 |
| AmcTmp | 0 | 0 | 0 | 0 | 1 | 0 |
| Amg | 9 | 9 | 2 | 5 | 0 | 2 |
| AmgAmc | 1 | 0 | 0 | 0 | 0 | 0 |
| AmgFqPhnSulTmp | 0 | 2 | 0 | 0 | 0 | 0 |
| AmgFqSul | 0 | 0 | 0 | 0 | 0 | 1 |
| AmgFqTetSulTmp | 2 | 4 | 0 | 1 | 0 | 1 |
| AmgPcn | 0 | 1 | 2 | 0 | 0 | 1 |
| AmgPcnAmcFqTetSulTmp | 1 | 0 | 0 | 0 | 0 | 0 |
| AmgPcnAmcTetSul | 0 | 1 | 0 | 0 | 0 | 0 |
| AmgPcnAmcTetSulTmp | 2 | 1 | 0 | 0 | 0 | 0 |
| AmgPcnFqPhnTetSulTmp | 1 | 0 | 0 | 0 | 0 | 0 |
| AmgPcnFqTet | 1 | 0 | 0 | 0 | 0 | 0 |
| AmgPcnFqTetSulTmp | 9 | 6 | 0 | 1 | 1 | 0 |
| AmgPcnPhnSulTmp | 6 | 0 | 0 | 0 | 0 | 0 |
| AmgPcnPhnTet | 1 | 0 | 0 | 0 | 0 | 0 |
| AmgPcnPhnTetSulTmp | 6 | 1 | 2 | 0 | 0 | 0 |
| AmgPcnSul | 5 | 3 | 0 | 0 | 0 | 0 |
| AmgPcnSulTmp | 11 | 1 | 1 | 1 | 0 | 1 |
| AmgPcnTet | 2 | 4 | 0 | 1 | 1 | 0 |
| AmgPcnTetSul | 3 | 3 | 1 | 0 | 0 | 1 |
| AmgPcnTetSulTmp | 47 | 40 | 4 | 10 | 13 | 2 |
| AmgPhnTetSulTmp | 2 | 1 | 0 | 0 | 0 | 0 |
| AmgPhnTmp | 1 | 0 | 0 | 0 | 0 | 0 |
| AmgSul | 6 | 2 | 0 | 1 | 0 | 0 |
| AmgSulTmp | 5 | 2 | 0 | 2 | 0 | 0 |
| AmgTet | 3 | 3 | 1 | 0 | 0 | 0 |
| AmgTetSul | 7 | 6 | 1 | 3 | 3 | 0 |
| AmgTetSulTmp | 13 | 20 | 0 | 2 | 1 | 1 |
| AmgTetTmp | 0 | 1 | 0 | 0 | 0 | 0 |
| AmgTmp | 3 | 0 | 0 | 0 | 0 | 1 |
| Ceph | 0 | 1 | 0 | 1 | 0 | 0 |
| CephAmg | 0 | 1 | 0 | 0 | 0 | 0 |
| CephAmgFqPhnSulTmp | 0 | 1 | 0 | 0 | 0 | 0 |
| CephAmgFqPhnTetSulTmp | 0 | 1 | 0 | 0 | 0 | 0 |
| CephAmgFqTetSul | 0 | 0 | 0 | 1 | 0 | 0 |
| CephAmgPcn | 0 | 0 | 1 | 0 | 0 | 0 |
| CephAmgPcnAmcTetSulTmp | 1 | 0 | 0 | 0 | 0 | 0 |
| CephAmgPcnFqPhnTetSulTmp | 0 | 1 | 0 | 0 | 0 | 0 |
| CephAmgPcnFqSulTmp | 1 | 0 | 0 | 0 | 0 | 0 |
| CephAmgPcnFqTetSulTmp | 2 | 1 | 0 | 0 | 0 | 0 |
| CephAmgPcnPhnTetSul | 0 | 1 | 0 | 0 | 0 | 0 |

| **Antibiogram** | **Human** | **Poultry** | **Bovine** | **Goat** | **Pig** | **Rabbit** |
| --- | --- | --- | --- | --- | --- | --- |
| CephAmgPcnTetSul | 0 | 1 | 0 | 0 | 0 | 0 |
| CephAmgPcnTetSulTmp | 2 | 4 | 1 | 0 | 1 | 0 |
| CephAmgTet | 0 | 0 | 0 | 1 | 0 | 0 |
| CephAmgTetSulTmp | 0 | 3 | 0 | 0 | 0 | 0 |
| CephPcnAmcFqTetTmp | 1 | 0 | 0 | 0 | 0 | 0 |
| CephPcnFqPhnSulTmp | 1 | 0 | 0 | 0 | 0 | 0 |
| CephPcnFqTetSulTmp | 1 | 0 | 0 | 0 | 0 | 0 |
| CephPcnSulTmp | 0 | 0 | 0 | 1 | 0 | 0 |
| CephPcnTetSulTmp | 0 | 0 | 0 | 1 | 0 | 0 |
| CephSul | 0 | 1 | 0 | 0 | 0 | 0 |
| CephTetSulTmp | 0 | 2 | 1 | 0 | 0 | 0 |
| Fq | 3 | 1 | 0 | 0 | 0 | 0 |
| FqSul | 0 | 1 | 0 | 0 | 0 | 0 |
| FqSulTmp | 1 | 2 | 0 | 1 | 0 | 0 |
| FqTet | 0 | 0 | 0 | 0 | 1 | 0 |
| FqTetSul | 1 | 0 | 0 | 0 | 0 | 0 |
| FqTetSulTmp | 2 | 4 | 0 | 0 | 0 | 1 |
| FqTmp | 1 | 0 | 0 | 0 | 0 | 0 |
| Pcn | 2 | 1 | 0 | 2 | 2 | 0 |
| PcnAmc | 1 | 0 | 0 | 0 | 0 | 0 |
| PcnAmcFqTetSulTmp | 0 | 0 | 0 | 0 | 0 | 1 |
| PcnAmcTetSulTmp | 1 | 0 | 0 | 0 | 0 | 0 |
| PcnFq | 1 | 0 | 0 | 0 | 0 | 0 |
| PcnFqTetSulTmp | 2 | 0 | 0 | 0 | 0 | 0 |
| PcnPhnTet | 1 | 0 | 0 | 1 | 0 | 0 |
| PcnPhnTetSulTmp | 1 | 3 | 0 | 0 | 0 | 0 |
| PcnSul | 0 | 1 | 0 | 0 | 0 | 0 |
| PcnSulTmp | 6 | 3 | 0 | 0 | 0 | 0 |
| PcnTet | 0 | 2 | 0 | 2 | 0 | 0 |
| PcnTetSul | 2 | 1 | 0 | 0 | 0 | 0 |
| PcnTetSulTmp | 9 | 13 | 2 | 2 | 3 | 1 |
| PcnTetTmp | 1 | 0 | 0 | 0 | 0 | 0 |
| Phn | 0 | 0 | 0 | 0 | 0 | 1 |
| PhnSul | 0 | 1 | 0 | 0 | 0 | 0 |
| PhnSulTmp | 1 | 0 | 0 | 1 | 0 | 0 |
| Sul | 18 | 18 | 3 | 11 | 1 | 4 |
| SulTmp | 23 | 17 | 3 | 0 | 0 | 0 |
| Tet | 7 | 12 | 0 | 2 | 0 | 0 |
| TetSul | 0 | 6 | 1 | 1 | 0 | 0 |
| TetSulTmp | 11 | 51 | 4 | 5 | 5 | 2 |
| TetTmp | 1 | 1 | 0 | 0 | 0 | 1 |
| Tmp | 2 | 2 | 0 | 0 | 0 | 0 |

Profile composition and number of *E. coli* isolates from human and animal in Nairobi, Kenya. Abbreviations: Cephalosporins (Ceph), Aminoglycosides (Amg), Phenicols (Phn), Tetracyclines (Tet), Penicillins (Pcn), Penicillin lactams (Amc), Folate inhibitors (Tmp), Sulfonamides (Sul) and Fluoroquinolones (Fq).

**Supplementary Methodological Appendix**

**Study Design**

The UrbanZoo project, funded by the Medical Research Council, aimed to understand the mechanisms of disease introduction and emergence in human populations in a major developing city, Nairobi, Kenya, in the context of livestock keeping. A significant component of the UrbanZoo project was the ’99 household project’ which focused on sampling of households across socio-economic strata of Nairobi to investigate the role of informal livestock keeping practices as a route of zoonotic disease emergence in humans.

As such, a cross-sectional study targeting sympatric human and livestock populations in Nairobi, was carried out from August 2015 to October 2016. Geospatial mapping data produced by Institut Français de Recherche en Afrique (IFRA) was used to identify 17 classes of residential neighbourhood in Nairobi based on different land-use patterns (e.g. residential, industrial, institutional) and physical landscape attributes (such as tree cover, plot size, amount of gated space, roofing type, presence of agriculture) (1). Subsequently, the 17 classes of neighbourhoods were ranked by average income and merged into seven wealth groups. Administrative sublocations were mapped onto each wealth group, identifying a total of 70 possible sublocations, for which dominant wealth groups were calculated by extracting the proportion of population belonging to each neighbourhood class within the sub-location boundaries. A total of 33 sublocations were selected to be included in the study, with the number of sublocations belonging to each wealth group chosen proportionately to the population density and the variety of neighbourhood classes in each of the seven wealth groups. Final selection of individual sublocations was aimed at maximising spatial distribution, socio-economic diversity, whilst attempting to capture the diversity of livestock keeping practices across the city (2).

For each sublocation, three geographical points were selected at random within the dominant housing type, comprising: two livestock keeping and one non-livestock keeping household. A total of 99 households, 66 of which kept livestock were visited. Livestock keeping households had to meet strict inclusion criteria of: (i) keeping small livestock only (small ruminants - goats/sheep, small monogastrics - poultry/rabbits), and (ii) large livestock (large ruminants (cattle), large monogastrics (pigs), with or without small livestock (Table s5). To ensure an equal sample of both cattle and pig-keeping households, the combination of livestock keeping households represented in each sublocation was randomised, and had to consist of either large ruminant and small monogastric, or large monogastric and small ruminant species. For sublocations in which households keeping large ruminant or large monogastric species were absent, a replacement household keeping either small monogastic or small ruminant species was recruited.

Within the sublocations, local administrative leaders assisted in recruitment, which was carried out a few days before the sampling date. The three pre-selected geographical points were identified on the ground, and the nearest three households that met the inclusion criteria identified.

**Table S5.** Criteria used in defining livestock keeping households

|  | **Large ruminant** | **Large monogastric** | **Small ruminant** | **Small monogastric** |
| --- | --- | --- | --- | --- |
| **Necessary and sufficient** | Cattle | Pigs | Goats/sheep | Poultry or rabbits |
| **Optional** | Any other species | Any other species | Pigs, poultry or rabbits | Cattle, sheep or goats |
| **Exclusion** | None | None | Cattle | Pigs |

**Sampling framework**

In each household, the household head/owner (or a nominated member) completed a questionnaire, detailing livestock ownership (e.g. abundance of livestock species), management practices (e.g. manure disposal practices), household composition (e.g. number of occupants), and socio-economic variables.

Thereafter, following an informed consent, every human member of the household was invited to contribute a faecal sample and answer questionnaires on: their age and gender, food consumption and medical history. Rectal swabs were obtained from (up to 20) livestock species present in the household (ensuring that all species were represented). Project clinicians, and veterinarians collected human and livestock faecal samples respectively. Questionnaires and data associated with samples was recorded using Open Data Kit (ODK) Collect software, on electronic tablets, and uploaded to databases held on servers at ILRI. Human and animal faecal samples were collected and transported on ice to one of two laboratories (University of Nairobi or Kenya Medical Research Institute) within 5 hours of collection.

**Statistical analysis**

Human population density was calculated as a proportion of the number of individuals per household. Household area was obtained by drawing the boundary of each household compound in ArcMap. Following recommendations by (3), initial data exploration was done to test for: (i) collinearity between explanatory variables, (ii) influence of outliers in the response or explanatory variables, and (iii) zero inflation (excess number of zeros). Box plots were used to visualise the median and the spread of the data. Where outliers in explanatory variables were present (e.g. count of livestock), log base 10 transformations were applied to the variable in question. To test for collinearity between explanatory covariates, for each model, relationships between all sets of explanatory covariates were assessed using multi-panel pairwise scatterplots, Pearson correlation coefficients, and variance inflation factors.

Optimal GLMM models were constructed based on hypothesis-driven biological reasoning, in which the understanding of the underlying system was taken into consideration. To determine whether excluding a variable increased or decreased model fit, we compared the full model to models with different variables removed using likelihood ratio tests in which the test variables were omitted from the full model by backward elimination of non-significant terms. All other control fixed effects and random effects were the same as in the full models. The importance of each variable was assessed by using a likelihood ratio test that compared the full model to a reduced model by dropping one test predictor. The fit of each model was reported as marginal regression coefficients of multiple determination (marginal R2). Model fit was checked by plotting the residuals versus fitted values. GLMMs were implemented in the R packages ‘lme4’ (4). The map of Nairobi was created using Geographic Information Systems (GIS) software ESRI ArcGIS105 version 10.4.1 (<http://www.esri.com/software/arcgis/arcgis-for-desktop>).

**References**

1. **Ledant M, Calas B, Fernandez R.** 2011. Socio-economical and infrastructural mapping and analysis of Nairobi. French Insitute for Research in Africa (IFRA), Nairobi.

2. **Bettridge JM, Robinson TR, Hassell JM, Kariuki S, Ward MJ, Woolhouse MEJ, Fèvre EM.** An epidemiologically structured sampling strategy to capture bacterial diversity in a changing urban environment, p. *In* (ed),

3. **Zuur AF, Ieno EN, Elphick CS.** 2010. A protocol for data exploration to avoid common statistical problems. Methods in Ecology and Evolution **1:**3-14.

4. **Bates D, Maechler M, Bolker B, Walker S.** 2014. lme4: Linear mixed-effects models using Eigen and S4. R package version **1:**1-23.
